# Bibliometrics for Social Validation

**DOI:** 10.1371/journal.pone.0168597

**Published:** 2016-12-22

**Authors:** Daniel J. Hicks

**Affiliations:** 1 Rotman Institute of Philosophy, University of Western Ontario, London, Ontario, Canada; 2 American Association for the Advancement of Science, Hosted in Office of Research and Development, United States Environmental Protection Agency, Washington, District of Columbia, United States of America; Beihang University, CHINA

## Abstract

This paper introduces a bibliometric, citation network-based method for assessing the social validation of novel research, and applies this method to the development of high-throughput toxicology research at the US Environmental Protection Agency. Social validation refers to the acceptance of novel research methods by a relevant scientific community; it is formally independent of the technical validation of methods, and is frequently studied in history, philosophy, and social studies of science using qualitative methods. The quantitative methods introduced here find that high-throughput toxicology methods are spread throughout a large and well-connected research community, which suggests high social validation. Further assessment of social validation involving mixed qualitative and quantitative methods are discussed in the conclusion.

## Introduction

The validation of novel scientific methods takes place at two levels. On the first level, or formal validation, methods are validated by showing that they are theoretically well-supported, their results can be replicated, they agree with established methods, and so on. The second level, or social validation, is the acceptance of methods by the relevant scientific community. Ideally, high formal validation would be both necessary and sufficient for novel methods to be broadly accepted by the scientific community. However, these two levels can operate independently. A novel method can have high formal validation when performed by its developers, but fail to be generally accepted by the scientific community. This might happen because the novel method is difficult to use, requires equipment or materials that are expensive or otherwise difficult to obtain, is very slow, or depends on assumptions that are not widely accepted or mathematical techniques that are not widely understood. Conversely, a method can become so widely adopted as the standard and expected technique in the field that it is applied and in ways that significantly weaken its formal validity. For example, [1] argues that this has happened with statistical hypothesis testing.

Social validation has been frequently studied in science and technology studies [STS] and philosophy of science for decades [2–5], primarily using qualitative methods. Fields such as bibliometrics, scientometrics, and meta-research [6] study scientific practice quantitatively. However, these fields either have not directly taken up questions of research validation (as in citation-based ranking studies) or have focused on formal validation (as in replicability studies). ([7] is a notable exception to this pattern.) This paper presents a quantitative method to study social validation using a citation network. As an illustration, the method is applied to the US Environmental Protection Agency’s high-throughput toxicology [HTT] research.

The US Environmental Protection Agency’s Chemical Safety for Sustainability [CSS] program is developing HTT data sources and analytical techniques to address limited toxicity and exposure data for tens of thousands of commercial chemicals [8]. Traditional toxicology methods focus on directly observable macrophysiological effects (e.g., tumors, death) in whole organisms (in vivo studies). By contrast, HTT methods focus on micro-level interactions (e.g., whether a chemical activates a certain cellular pathway) and use systems biology and computational simulations (in vitro and in silico studies).

For some particular applications, HTT methods have been shown to have degrees of formal validation comparable to those of traditional, in vivo methods [9]. In other cases the formal validation of currently-existing HTT methods is low [10, 11]. There has been no previous systematic study of the social validation of these methods.

## Methods

### Conceptual Model

**Fig 1:**
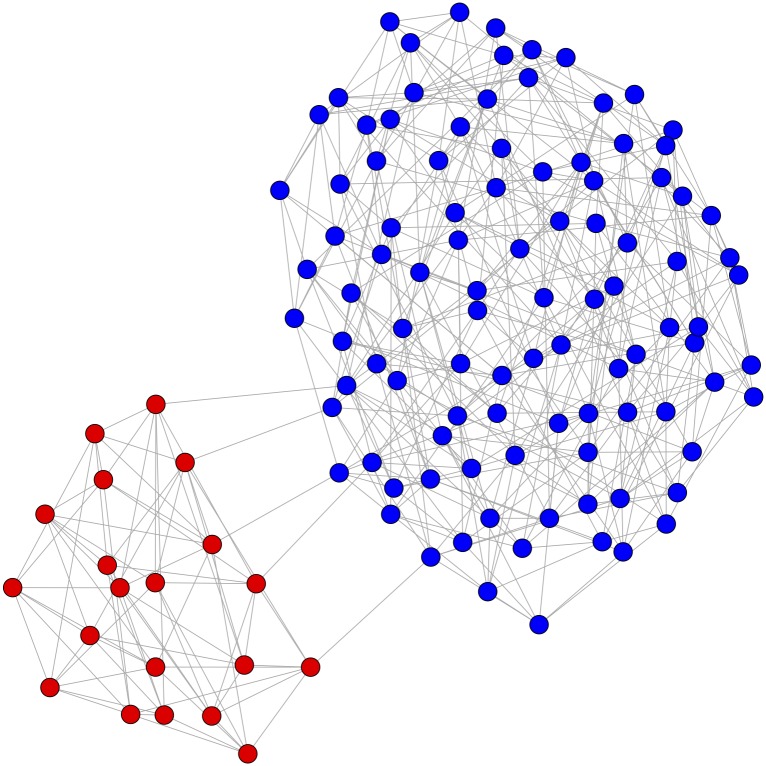
Hypothetical citation network indicating low social validation. Nodes represent publications, e.g., journal articles; two publications are linked if one publication cites the other. While the network is connected, red and blue nodes form distinct communities with only a few ties between them, suggesting the methods used by authors of the red publications could have low social validation.

Citation networks have been used in bibliometrics since the 1970s, and today are a standard tool of the field [12–16]. Fig 1 is a conceptual model of how citation networks can be used to quantitatively analyze social validation. In this network, nodes (also called vertices, and occasionally dots or points) are publications, and two publications are linked with an edge if one publication cites the other. Two communities—subsets of the entire network, indicated by blue and red coloring of the nodes—have been identified using a standard algorithm [17]. There are dense connections within these two communities, but relatively few connections between them; in other words, blue papers tend to cite other blue papers, and red papers tend to cite other red papers, but relatively few blue papers cite red papers and vice versa. Low social validation provides one possible explanation for this network structure; perhaps researchers publishing in the red community are using methods that are not generally accepted by their peers in the blue community.

The strength of a partition—a division of a network into mutually exclusive and jointly exhaustive communities—can be quantified using the modularity statistic [17], defined as

$$Q=\sum_{i=1}^{k}\left[\frac{e_{i}}{m}-{\left(\frac{d_{i}}{2m}\right)}^{2}\right],$$

where *Q* is the modularity statistic, *i* indexes the communities in a given partition of the network (in Fig 1, *k* = 2), *e*_*i*_ is the number of intracommunity edges in *i* (i.e., edges between two members of *i*), *d*_*i*_ is the total degree of all nodes in *i*, and *m* is the total number of edges in the network. *Q* is meant to capture the thought that a well-defined community has more intracommunity edges (corresponding to the term *e*_*i*_/*m*) than would be expected in a random rewiring of the network (corresponding to the term (*d*_*i*_/[2*m*])^2^).

**Fig 2:**
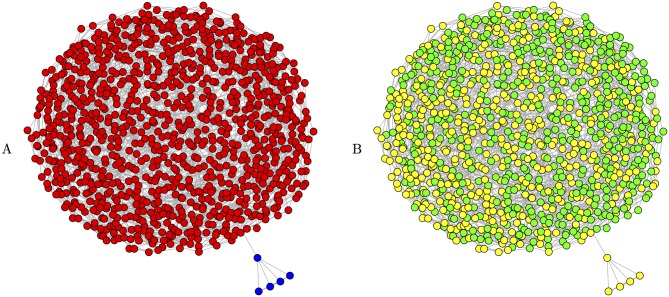
Modularity optimization can miss “intuitively modular structures”. Both subfigures show the same network, comprised of a small subnet of 5 nodes with a single link to a large subnet of 1,000 nodes. *A*: The network as constructed; small subnet nodes are blue and large subnet nodes are red. For this partition, *Q* ≈ .005, $S_{small}=\frac{10}{11}\approx .91$, $S_{large}=\frac{2000}{2001}\approx .995$. *B*: The result of a modularity-optimizing community detection algorithm, with the two “communities” in yellow and green. Note that the algorithm completely misses the constructed partition of small and large subnets. For this partition, *Q* ≈ .156.

Over all partitions of all networks, the value of *Q* ranges between $-\frac{1}{2}$ and 1, where greater values of *Q* are interpreted as a sharper division of the network into subcommunities [17]. However, on any given network, the maximum and minimum values of *Q* across all possible partitions can be a much smaller subset of this interval. For example, the partition in Fig 1 maximizes *Q* for this network at 0.16. Many algorithms for community detection try to identify partitions that maximize the value of *Q*. However, [18] shows that modularity optimization can be noisy and miss “intuitively modular structures,” especially in large networks. For instance, consider a nearly disconnected network built by connecting two regular graphs of degree 4, *G*_1_, *G*_2_, with a single edge (Fig 2). Suppose *e*_1_ ≪ *e*_2_ ≈ *m*, and consider the formula for *Q* on the partition corresponding to these connected components. Since $\frac{e_{1}}{m}\approx \frac{d_{1}}{2m}\approx 0$, in the formula for *Q* the term for *G*_1_ is approximately 0. And since $\frac{e_{2}}{m}\approx \frac{d_{2}}{m}\approx 1$, the term for *G*_2_ is also approximately 0. Thus, while “intuitively” the partition of the network into *G*_1_ and *G*_2_ should have high modularity, *Q* goes to 0 as the relative sizes of the components increase, and thus this partition is likely to be missed by a modularity optimization algorithm.

To avoid this problem, an insularity statistic is also calculated. Given a community *i* of a partition *s*, we define insularity as

$$S_{i}=\frac{e_{i}}{m_{i}},$$

where *S*_*i*_ is the insularity statistic, *e*_*i*_ is the number of intracommunity edges, and *m*_*i*_ is the number of community edges, i.e., edges with at least one end in the community. In the example of *G*_1_, *G*_2_ connected by a single edge, insularities are $S_{i}=\frac{e_{i}}{e_{i}+1}\approx 1$ as *e*_*i*_ increases.

**Fig 3:**
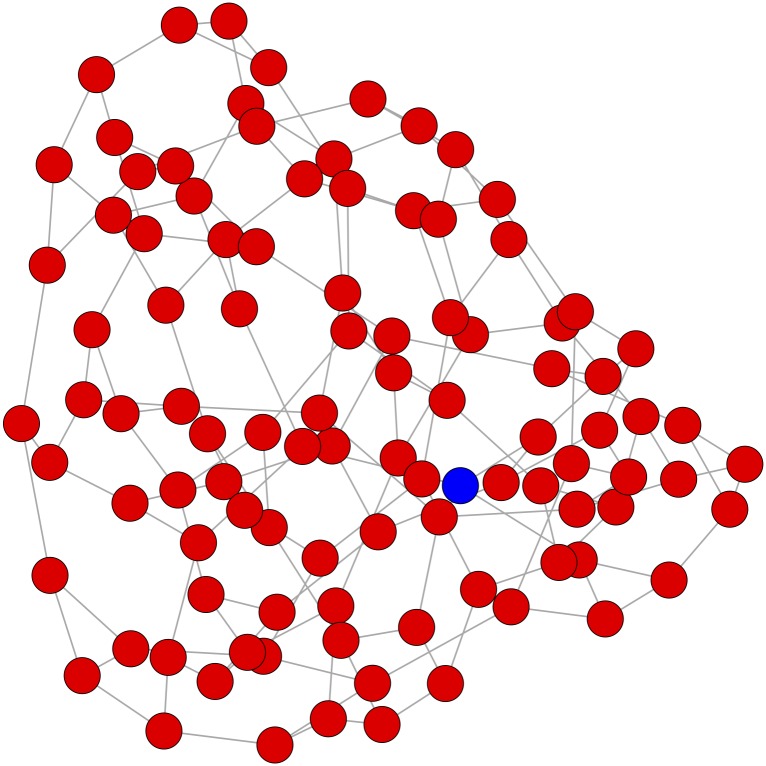
Insularity can be spuriously high with small subsets in much larger networks. A regular network of degree 3 on 100 nodes is generated, and partitioned by selecting one node (blue) uniformly at random. The insularity of the large subnet (red) is very high, $S=\frac{147}{150}=.98$, but the modularity of the partition is extremely small, *Q* ≈ .0001.

Informally, insularity is negatively associated with the ratio of “surface area” to “volume” (when insularity is high, this ratio is low). With a relatively small community in a large network, high insularity can indicate that the community is isolated from the rest of the network. At the same time, with a relatively large community, high insularity can simply reflect the large “volume”; see Fig 3. Consider a network with *n* ≫ 1 nodes and uniform degree *d*, and a community comprising every node except for one; this community will have $S=\frac{d(n-1)/2}{dn/2}=\frac{n-1}{n}\approx 1$.

These points suggest that *Q* and *S* can be highly informative, but must be interpreted with care.

### Network Construction

**Fig 4:**
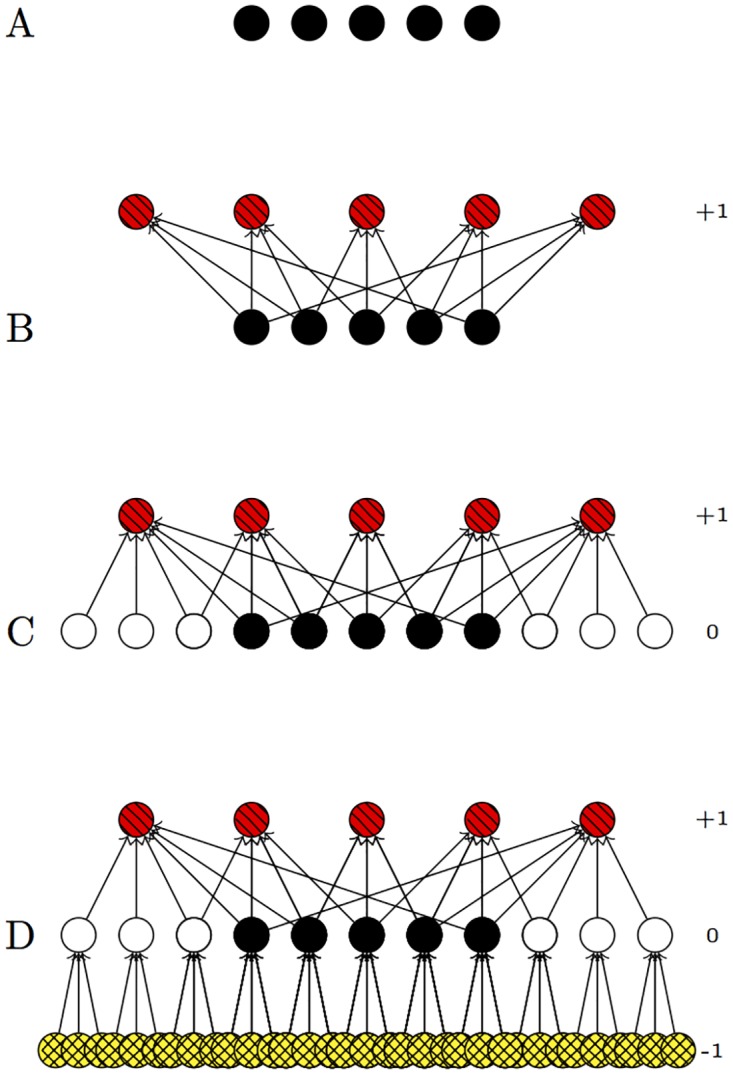
Conceptual model for the sequential construction of the citation network. *A*: Construction begins with a set of given or “core” publications (black). In the use case discussed below, these are publications by researchers in the CSS program. *B*: Downstream or forward citation search identifies other publications (red, diagonal pattern) that cite the core set. These are designated “generation +1.” *C*: Automated backwards citation search identifies publications (white and black) cited by the generation +1 publications. These are designated “generation 0,” and include the core set. *D*: Automated backwards citation search identifies publications (yellow, crosshatch pattern) cited by white and black publications, as “generation -1.”

The citation network is constructed iteratively using metadata retrieved from Elsevier Scopus using the Scopus API [application programming interface] and custom Python scripts. A conceptual model for the construction process is given in Fig 4. Complete source code and further discussion of the network construction scripts is available at [19], and a compressed GraphML file containing the citation network used in the case study is available at [20].

[16] finds that “bibliometric data from [Web of Science] or Scopus [are] adequate to conduct research evaluations” in physical and biological science ([16] p4), but that Google Scholar contains a significant number of errors, and that coverage of Web of Science and Scopus can be limited for other research fields. PubMed has limited bibliometric data, and has limited coverage outside of biomedical research. Both Web of Science and Scopus have APIs, which can be used to efficiently search and retrieve large amounts of data. All together, both Web of Science and Scopus are appropriate sources of data for this kind of analysis. However, I was unable to find useful documentation for the Web of Science API; by contrast, Scopus’ API is documented at http://dev.elsevier.com. For this purely practical reason, I chose to work with Scopus here.

[16] describes two methods to construct a citation network for a given author: a forward citation search on the author’s publications, and a backwards search for citations to the author across all publications in the research database. Another common method is based on the bibliographies of every article published in a given journal (or set of journals) over a period of time. The two methods described by [16] give a limited view of the relevant scientific community; and the journal-based method will miss citations coming from outside of the predetermined set of journals.

Given these limitations of previous methods for constructing a citation network, the method developed here involves a novel, iterative construction. See Fig 4. Construction begins with a set of given publications (in particular, a list of DOIs [digital object identifiers]). Given these “core” publications, Scopus’ web interface is used to manually conduct a forward or downstream citation search on the core set, retrieving every publication that cites a core publication. These publications are designated “generation +1.” A set of Python scripts then automatically retrieves metadata for generation +1, including bibliography contents; the papers cited by generation +1 become generation 0. Note that generation 0 should include every core publication. The Python scripts then retrieve the metadata for generation 0, and the papers cited by generation 0 form generation -1. The script included in the code repository then retrieves the metadata for generation -1. However, in the use case below, generation -1 contained over two million papers. Given weekly caps on the number of requests that can be sent to the Scopus API, retrieving the metadata for generation -1 would have taken several months. Thus, for the use case below, metadata retrieval stopped at generation 0.

This construction approach is somewhat similar to the method used by [21] to construct co-citation networks, which also involves a combination of forward and backward citation searches. However, in their construction, the reference article (equivalent to the core publications) is connected to every node in the resulting network. This method is therefore inappropriate to assessing social validation in terms of whether the articles of interest are isolated within one part of the citation network.

After retrieving the metadata, a small sample of metadata entries is extracted, allowing the analyst to manually check the quality of the metadata. The Python package graph-tool is then used to construct, manipulate, analyze, and visualize the citation network [22]. Connected components of the network are separated and analyzed as independent networks. For analysts interested in coauthor networks—in which the nodes are authors and two nodes are connected if, and only if, the two authors are listed as coauthors on at least one paper in the dataset—the code in the repository also constructs these kinds of networks, though they are not analyzed here. After analysis, copies of the networks are saved in gt and graphml formats; gt is a compact, binary format supported only by graph-tool, while graphml is a widely-supported xml-based format.

### Analysis

As discussed above, the two key statistics, *Q* and *S*, need to be interpreted with some care. In what follows, I refer to the partition of the citation network into core and non-core nodes as the core partition. The core partition has values for *Q* and *S*, but it is not clear what these statistics mean, taken in isolation. The analytical approach developed here places these statistics into context using up to 8 different analyses, which apply 3 analytical methods across 3 different networks.

#### Sampling Distributions

In the random sample method, *Q* and *S* are calculated for random sets of nodes (each with the same size as the core set); in the noisy community detection method, these statistics are calculated for binary partitions (that is, partitions with exactly two communities) identified using a modularity-maximization method. (Community detection is discussed in more detail below.) For both of these methods, by iterating the construction of the partition several hundred times, I build sampling distributions for *Q* and *S*, then calculate a *p*-value for the observed values of *Q* and *S* for the core partition against this sampling distribution, asking “how frequently did values at least as extreme as the observed values occur?” Since the core set is known not to be constructed using a random sample or modularity optimization, this *p*-value should not be interpreted as a likelihood in a hypothesis test. But it can be used, as in a hypothesis test, as an indicator of the “distance” between the core set and some hypothetical constructions. For example, *p* = .3 for the random sample method might be interpreted as indicating that the core set is distributed *as though* it were a random set of nodes, while *p* = .002 for the noisy communication detection method might be interpreted as indicating that the core partition is not distributed *as though* it were a modularity-maximizing partition.

These 2 sampling distribution-based analyses are applied to 3 different networks: the constructed citation network, as well as two reference networks from the high-energy physics and theoretical physics communities of the arXiv online repository [23, 24]. Comparison to reference networks could allow us to detect unusual features of the constructed citation network. These particular reference networks were chosen as publicly available and widely-used models of citation networks.

#### Noisy and Stable Community Detection

In addition to the problems of missing intuitive structure and returning spurious structure, modularity optimization can be highly noisy, in the sense of producing dramatically different partitions of a given network with approximately the same maximum modularity [18]. I therefore refer to this as noisy community detection. (For a comprehensive review of community detection methods, see [25].)

Given the problems with noisy community detection, in this paper it is not considered a reliable way to identify structure in the citation network. However, noisy community detection can still be considered a reliable way to discover the “modularity plateau” of partitions that approximately maximize modularity. Comparing the modularity values for these partitions to that of the core partition can tell us whether the core partition is within, or close to, that modularity plateau. In graph-tool, noisy community detection is implemented by an algorithm that attempts to optimize modularity using a statistical mechanics approach [26].

graph-tool also implements an alternative approach to community detection, referred to as a stochastic blockmodel approach [27, 28]. A blockmodel is a generative model for networks: the model assumes a set of discrete blocks (that is, communities) and edge frequencies within and between the blocks; a given network is generated from the model by randomly adding edges according to the edge frequencies. Given a block set (that is, partition) **b** and network *G*, model fit is calculated as the posterior probability *pr*(**b**|*G*) using Bayes’ theorem:

$$pr\left(b|G\right)=\frac{\sum_{\theta }pr\left(G|\theta ,b\right)pr\left(\theta ,b\right)}{pr(G)},$$

where *θ* are other model parameters. Maximizing this posterior probability is equivalent to minimizing the description length

Σ=-lnpr(G|θ,b)-lnpr(θ,b),

which can be interpreted information-theoretically as the amount of information required to describe the network *G* given *θ* and **b**.

While this algorithm is not deterministic, the resulting values of *Q* and *S* do not vary enough to apply the sampling distribution analysis. I therefore refer to it as stable community detection. Instead of constructing a sampling distribution, a contingency table is used to compare the communities detected by one run of this algorithm (set to produce a binary partition with exactly two communities) against the core partition. Since there is no meaningful way to compare the results of this algorithm when it is applied to different networks, this analytical method was not used with the two physics reference networks.

### Data Validation and Robustness Analysis

Like other online research databases, Scopus does not have complete coverage of all academic journals and other publishing venues, and can potentially have errors in its individual records. The network construction method introduces further possibilities for data error.

To validate the data, the scripts generate a tractable sample of 100 DOIs from the final dataset. The analyst can then manually check key pieces of information, such as the number of bibliography entries, comparing the dataset to the publication version of record.

The modularity and insularity statistics depend on the network topology, which cannot easily be checked by examining a small number of individual records. However, two methods of robustness analysis can be used. First, the analysis can be repeated under varying inclusion/exclusion criteria. In the use case below, in the primary run, the analysis examined only publications dated 2006 or later; then in a secondary run the analysis was restricted to publications dated 2011 or later. An alternative approach would randomly rewire a fraction of the edges in the network, such as 10%, to simulate the effects of data errors on the network topology. This approach would be more useful for smaller or less densely connected networks than the one examined in the use case, since these networks will be more sensitive to rewiring.

### Text Analysis

Because network community detection is based purely on the topology of the network, the resulting communities can be difficult to interpret. Similarly, the layouts used to visualize networks are generally based purely on network topology; while they may suggest communities and other structures, they can also be difficult to interpret. Pairing network topology-based analyses with text analysis might support meaningful interpretations of community memberships or other features of the network structure.

Different functions in the Scopus API return different sets of article data. The particular function used in the code developed for this project includes both backwards citations (reference lists) and abstract text for articles, but does not include article fulltext. Other functions include article fulltext, but these do not include backwards citations in a convenient format. Storing article text, whether abstracts or fulltext, also significantly increases the storage and memory resources required to work with these networks.

For these practical reasons, the primary code developed for this project does not store article abstracts, and so does not directly support text analysis. However, an exploratory analysis of the core paper abstracts alone is much more tractable, and potentially informative. Such an analysis is conducted in S1 Topic Analysis of the Core Set Abstracts. In addition, at the end of the use case I discuss several ways in which, given sufficient computational resources, more sophisticated text analysis techniques could be applied to the question of social validation.

## Use Case: Social Validation of HTT

As discussed in the introduction, the USEPA’s Chemical Safety for Sustainability [CSS] program is developing novel methods for testing chemical safety. The citation network methods described in the previous section were developed to investigate the social validation of these novel methods within the broader research community. A list of CSS publication titles was retrieved from STICS, a USEPA-internal database of research products; DOIs were identified for these publications using semi-automated title searches. The CSS publications for which DOIs were found formed the core publications for the citation network (see the explanation of the construction method in Fig 4).

**Fig 5:**
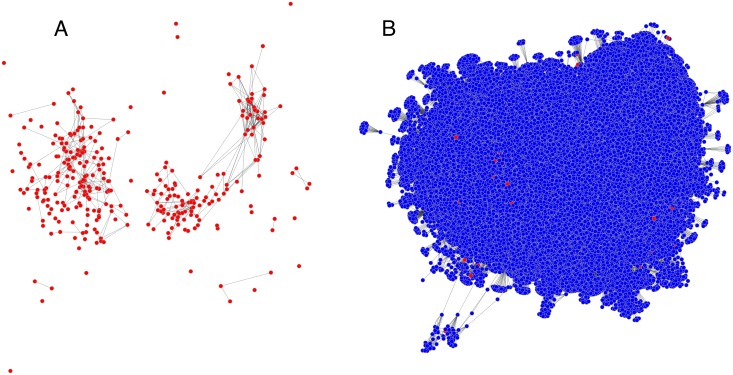
Citation network for high-throughput toxicology research. *A*: The core nodes alone. *B*: The full citation network of 80 thousand nodes. Positions in the two panels roughly correspond.

The citation network for CSS research is shown in Fig 5, using a publication cutoff of 2006. This network comprises 80.1 thousand nodes connected by 663 thousand edges, including 323 core publications. Although the core publications are highly disconnected, the full network is connected. This connectivity was not expected; CSS researchers work at several different USEPA national labs and centers, on a diverse set of topics, and using a wide range of methods. When a publication cutoff of 2011 is used instead, the network has 48.5 thousand nodes, 214 thousand edges, all 323 of the CSS publications, and is still connected.

The core partition for this network has modularity *Q* = 9.5 × 10^−4^, and the core nodes have insularity *S* = .032; that is, only about 3% of their edges are shown in the right panel of Fig 5. In random samples, the mean values of modularity and insularity were *Q* = 3.0 × 10^−9^, *S* = .0020. With the noisy community detection algorithm, the mean sample modularity was *Q* = .42 and the mean insularity was *S* = .86. In all of these analyses, *p* = 0; that is, neither random sampling nor noisy community detection produced any values as extreme as the observed values for *Q* and *S*. Using the 2011 cutoff, *Q* = .0028, *S* = .052; random samples had *Q* = 5.3 × 10^−7^, *S* = .0034; noisy communication detection had *Q* = .41, *S* = .84; and with all of these sampling distributions *p* = 0.

These statistics indicate that CSS publications have very low modularity and insularity, and are not distributed as though they were either a random subset or a modularity-maximizing partition.

Both of the reference networks had on the order of 30 thousand nodes and 400 thousand edges; these values were on the same order of magnitude as the constructed network, and the constructed network was larger in terms of both nodes and edges. However, the reference networks had relatively more edges. (Edge:node ratio: constructed network: 8; reference networks: 13.) In the reference networks, for the random partitions, modularities were on the order of −1 × 10^−6^ and −7 × 10^−7^ and insularities were approximately .002. As in the constructed network, in all of these analyses *p* = 0. Since the noisy community detection algorithm is quite slow (500 runs of the algorithm took approximately five hours for the constructed network on available computing equipment), that comparison was skipped for this use case.

Again, these statistics indicate that the core publication’s modularity and insularity were low, though not as low as a random sample of nodes.

**Fig 6:**
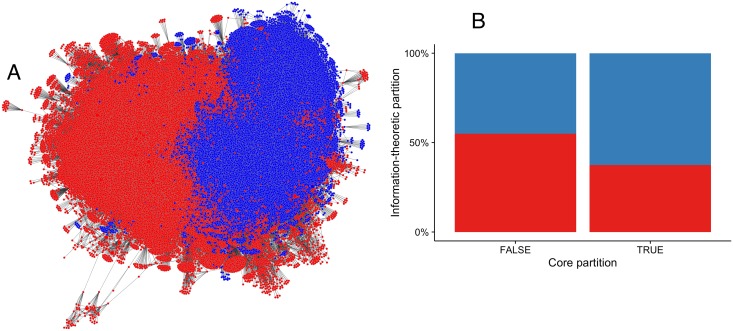
Comparison of core and blockmodel partitions of the citation network. *A*: The citation network, using the same positions as in Fig 5. Blockmodel partition indicated by node color. Core publications are indicated by larger nodes. *B*: Percentage distribution of core and other nodes across the two blockmodel communities. Bars correspond to core (right) and other nodes (left). Blockmodel partition is indicated by node color. Note that the y-axis is a percentage scale, not an absolute node count. Compare to Table 1.

The partition produced by the stable, blockmodel community detection method is shown in Fig 6, and a contingency table for the partitions is given in Table 1. This partition has *Q* = .44 and insularities of .97 and .95. Modularity is more than three orders of magnitude larger than the modularity for the core partition. Insularities are also very high, although this should be interpreted with caution; as noted above, with large partitions, large insularities reflect the low “surface area” to “volume” ratio. Note that these values are similar to those produced by the noisy community detection algorithm. When the 2011 threshold is used instead, these values are only trivially different: *Q* = .45, *S* = .94, .92.

**Table 1:** Contingency table for the core and blockmodel partitions. Counts (row-wise percentages). Compare to Fig 6B. *χ*^2^ = 40, *p* = 3.0 × 10^−10^, *V* = .022.

	Community 0	Community 1
**CSS**	121 (37%)	202 (63%)
**non-CSS**	43,839 (55%)	35,958 (45%)

Within the partition produced by this stable community detection algorithm, CSS nodes are 33% less likely to be in Community 0 than non-CSS nodes, and a *χ*^2^ test for independence on this table is conventionally highly statistically significant (*p* = 3.0 × 10^−10^). However the effect size—as measured by Cramér’s *V*, a correlation coefficient—is conventionally small and—given a significance level of 5%—this *χ*^2^ test reaches 99% power at an effect size of just .015, suggesting that it could be overpowered for this case [29, sec. 5.4]. These findings are approximately the same when the 2011 threshold is used instead.

All together, this analysis has the following key findings for the case of CSS research:

- CSS publications appear in a single large, connected citation network.
- CSS publications have very low modularity and insularity, indicating that they are not isolated within the citation network.
- CSS publications are not distributed evenly across the communities of the network, or as though they were a random subset, though this bias is small.
- All together, CSS publications are well integrated into the research community.
- The above findings are robust when 2011 is used as the publication date threshold rather than 2006.

These findings provide evidence that CSS research enjoys high social validation.

### Further Assessment of Social Validation

The analytical approach presented in this paper could be extended in several ways in future work. The CSS program is divided into four different topics and several different projects. These divisions could be used for a more complex, community-based analysis, i.e., the integration and distribution of these four topics across the citation network. Unfortunately, in this project there was no efficient way to associate individual papers with their USEPA topics or projects.

This paper focuses on publications using a citation network. The Python code developed for this project also constructs and analyzes coauthor networks. For CSS, the findings of this coauthor network analysis were qualitatively similar to the citation network findings reported above. However, for other cases the coauthor network analysis could be more relevant, or notably disagree with the citation network analysis.

Further use of community detection algorithms could also be useful in some cases. For example, the stable community detection algorithm could be applied (without the limitation to produce a binary partition) to both the core nodes and the entire citation network, and membership in the two partitions could be compared. This might reveal, for instance, that the core nodes enjoy high social validation in some parts of the literature but low social validation in others. Because the CSS nodes taken by themselves were highly disconnected, this algorithm did not find any further structure in the core nodes, and thus this use of community detection was not applicable to the case examined in this paper.

As discussed above, for practical reasons the primary code developed for this project does not store article abstracts or other text data. Extensions to the code, supported by sufficient computational resources, could be used to conduct more sophisticated text analysis. [30] develops a notable approach based on topic modeling. Briefly, topic models use word co-occurrence patterns and a generative model of text construction to identify latent topics or themes across a collection of texts. (Topic models are used in the exploratory text analysis included in S1 Topic Analysis of the Core Set Abstracts.) While topic model algorithms are essentially syntactical, human reviewers of the resulting topics often find them meaningful and semantically rich [31]. [30] extends the generative model to a citation network; a given text is modeled as incorporating the topics found in the upstream texts that it cites. Further, the extent to which the distribution of topics across a given text resembles those of its predecessors supports a quantifiable estimate of the influence of each predecessor text. This method could provide a rich analysis of social validation across the citation network. Higher influence means that the downstream text is using language that is distinctive to the upstream text. This language use can then be interpreted as an indication of the extent to which the downstream authors accept the novel methods and conceptual frameworks of the upstream text. Given the complexity of the approach presented in [30], it was not attempted here.

[32] describes a method using natural language processing to characterize citations as “positive” or “negative.” This kind of analysis could be extremely valuable for assessments of social validation; the findings discussed in the last section indicate that CSS research is integrated into the research community, but perhaps much of the discussion of it is negative. However, the method described in [32] involved a team of 5 researchers manually constructing a training set of 15,000 citations. Given limited resources, this method was not applied here.

A tractable sample of 25 generation +1 papers was drawn, and the citations to core papers were examined manually. These generation +1 papers cited 30 core papers. For 2 of the generation +1 papers papers, fulltext was not available, and so the citations could not be examined. 23 of the remaining 28 citations were classified as “point citations,” a single citation to the core paper as the source of a value, description of an experimental method, etc. A further 3 citations were classified as “repeated point citations,” in which the core paper was referred to more than once (in the given generation +1 paper), but each time as a source of a value, description of an experimental method, or other point-like reference. 1 generation +1 paper referred to the core paper as a “companion paper” by the same authors, and 1 generation +1 paper gave a paragraph-length discussion of the cited core paper. None of the 28 available citations were considered negative.

This small sample suggests that, while CSS research is well-integrated to the scientific community, a typical citation to a CSS publication treats it as “just another data point,” rather than a radically new approach. However, it could be that the typical CSS publication is only meant to report “just another data point,” rather than present HTT as a radical new approach to toxicology. In Kuhnian terms, perhaps a typical CSS publication is “normal science,” and only a few papers are intended to announce “revolutionary science” [3]. In this case, it would be useful to focus on citations to a subset of CSS publications that are written as “calls to revolution,” such as programmatic papers or high-level reviews.

## Conclusion: Further Assessment of Social Validation

This paper has introduced a method for quantitatively assessing the social validation of a body of research using an iteratively-constructed citation network. This method was applied to the case of high-throughput toxicology research, showing that this research enjoys high social validation.

## Supporting Information

S1 Topic Analysis of the Core Set Abstracts Text analysis of the core set. (PDF)
