# Supplementary material for: Bibliometrics for Social Validation
Source: PLoS One. 2016 Dec 22;11(12):e0168597. doi: 10.1371/journal.pone.0168597 (PMC5179025; doi:10.1371/journal.pone.0168597)
Supplement: S1 Topic Analysis of the Core Set Abstracts — (PDF) [file pone.0168597.s001.pdf]

S1 Topic Analysis of the Core Set Abstracts

Daniel J. Hicks

11/15/2016

Contents

Introduction **1**

Data **1**

Methods **2**
Topic Stability 2
Topic-Partition Comparison 2

Results **3**

Conclusion **6**

Appendix: Reproducibility **6**

References **7**

Introduction

Text analysis could be used to interpret the blockmodel partition in figure 6 of the main paper. To be clear, this partition is imposed on the citation network, in the sense that the partition algorithm is forced to produce a partition with exactly 2 communities. 2 communities would not necessarily minimize the description length of the network, and thus might not be optimal from the perspective of the blockmodel approach. For the purposes of this project, the optimal partition structure of the citation network is less interesting than whether the core vertices are confined to a single community of a binary partition of the whole network.

However, text analysis might still be useful for understanding the core set itself. Specifically, does the blockmodel partition of the core set correspond to any textual differences? This analysis examines that question. For data reasons discussed below, as well as considerations of length, this analysis was conducted in an exploratory fashion, rather than integrated into the primary analysis discussed in the main paper.

Data

Paper abstracts were not retrieved in the original data collection process. This was primarily a matter of computational resources. Due to the number of nodes of the network, even with only relatively little paper metadata, the `graphml` file containing the network is relatively

large (nearly 1 GB on disk). Including abstracts could easily increase the size of this file 2 or 3 times. Working with a single file this large could have severely taxed the computational machinery available for this project; and distributing the data across multiple files would have significantly complicated project file management and downstream analysis. Also, due to weekly quotas imposed by the Scopus API, retrieving abstracts for 80,120 papers would have delayed revisions to the manuscript by several weeks. By contrast, text analysis of the core set papers alone would require far fewer resources, and abstracts for all of these papers can be retrieved in less than an hour. For these reasons, I focus here on a text analysis of the core set abstracts.

Methods

Topic models, fit using Latent Dirichlet Allocation, are a common tool in unsupervised text analysis, even in fields that do not traditionally use computational methods [1,2]. Briefly, topic models use a Bayesian method to group words into a given number of topics based on their co-occurrence patterns. Documents are modeled as drawing words randomly from these topics according to a latent conditional distribution across topics: $\gamma_{i,j} = \mathrm{pr}(\mathrm{topic}_i|\mathrm{document}_j)$. A high value of $\gamma_{i,j}$ indicates that document j draws almost exclusively from topic i. Topic models have been found to be efficient for discovering syntactic patterns across large-document corpuses, even when each individual document is small, such as Twitter tweets [3].

Topic Stability

Topic model algorithms generally require analysts to manually specify the number of topics k in the model, and fitted models are generally evaluated either by their ability to classify held-out documents according to human-curated categories [4] or by human judgment that the word assignments to topics are "relevant and intuitive" [5]. By contrast, [6] provide a method to quantitatively assess the stability of topic modeling across a range of values of k. Briefly, the method first constructs several subsamples $s_1, \ldots, s_n$ of the entire corpus; below, we use 50 samples, each comprising 80% of the documents in the core set. Next, for each value of k under consideration, the method fits a model with k topics to the entire corpus s_0 and the subsamples $s_1, \ldots, s_n$. An agreement score is then calculated for each $s_1, \ldots, s_n$, relative to s_0. The distribution of agreement scores for a given value of k indicate the stability of the topic model with k topics for the entire corpus s_0.

The method introduced by [6] evaluates agreement in terms of the rank lists of terms in each topic model; roughly, two models agree insofar as the top 20 terms in each topic are the same. For the purposes of the current analysis, the stability of the document assignments is more interesting than the stability of the term lists. Thus, in this analysis, agreement scores between models are calculated in terms of the correlations of the $\gamma_{i,j}$, the posterior distributions of topics for each document.

Topic-Partition Comparison

After a value of k is selected, we compare the document assignments in the topic model to the blockmodel partition. Because topic models fit posterior probabilities $\gamma_{i,j}$, topic assignment

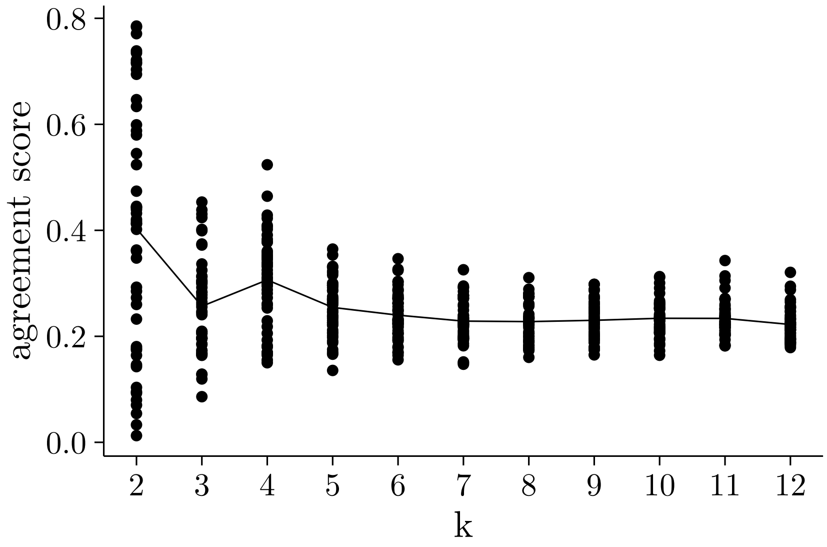

Figure S.1: LDA stability analysis results. Points report agreement scores for corpus subsamples fit to a topic model with k topics. The line gives the mean agreement at each value of k.

can be handled either discretely or continuously. For a discrete assignment, document j is assigned to topic i if, and only if, $\gamma_{i,j}$ is greater than a certain threshold; below we use $\gamma_{i,j} > .8$. With $k = 2$ topics, this gives us 3 bins of core papers: in topic A, in topic B, and in neither topic. This tripartite classification of papers is then compared to the blockmodel partition assignments, using a contingency table approach similar to that used to compare the core partition with the blockmodel partition.

With $k = 2$, topic assignments can be handled continuously by simply working with one of the two families $\gamma_{A,j}$, that is, the posterior probabilities of a given topic A across all documents j. Since $\gamma_{A,j} = 1 - \gamma_{B,j}$, low values of $\gamma_{A,j}$ correspond to documents j that are "assigned" to topic B.

Results

In figure S.1, points indicate the agreement score for each sample at each value of k; the line indicates the mean agreement score at each value of k. The plot indicates that $k = 2$ is somewhat more stable than other values of k, on average, but with much more variance. Thus, $k = 2$ should be considered at best only moderately stable. We proceed with $k = 2$ as the best available option.

The network contains 323 core nodes, but Scopus returned abstracts for only 319 papers. For discrete topic assignments, documents — core paper abstracts — are binned into topics using a threshold $\gamma_{i,j} > .8$.

To visualize the distribution of topics over the core nodes, we work with the continuous γ value for one of the two topics, coloring nodes more red insofar as they have a higher

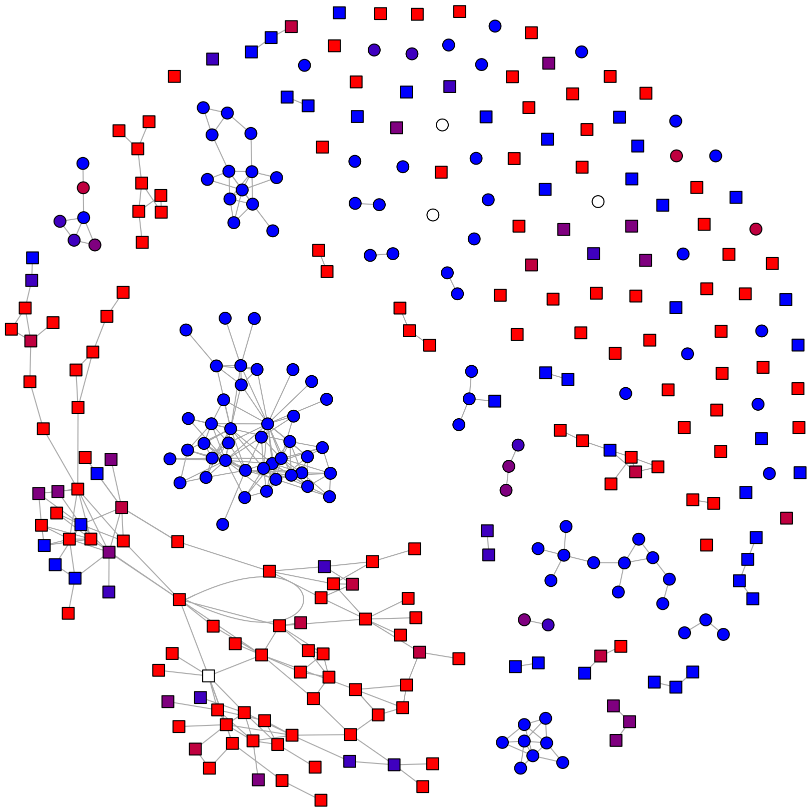

Figure S.2: The core nodes. Node shading indicates topic assignment; papers fully associated with topics are either pure blue or pure red, and papers in between are shades of purple. Node shape indicates blockmodel partition membership; circle nodes are in partition 0, while square nodes are in partition 1. Note that `igraph`, used for network analysis and plotting in this supplement, cannot interpret the layout calculated by `graph-tool`. Therefore the positions in this plot do not correspond to those in the figures in the manuscript.

posterior value for this topic and more blue insofar as they have a lower value. Node shapes are used to indicate blockmodel community membership. See figure S.2.

The connected components are generally homogeneous in terms of topics, though the largest connected component contains several purple nodes — indicating a mid-range value of γ — and a few blue ones. To investigate this further, we plot the γ values of a given node's neighbors against the node's own value of γ; see figure S.3.

In figure S.3, the blue line indicates a linear regression. This regression fits the data much better than might be suggested by the plot; $R^2 = 0.73$. (The appearance of poor fit might be due to tightly-clustered points near the upper-right and lower-left corners of the plot.) This strong correlation indicates that connected nodes tend to have very similar topic assignments.

In the network plot, different node shapes correspond to the two communities of the blockmodel partition. This visualization strongly suggests a correlation between topics and this partition. To make this comparison more carefully, we construct a contingency table of

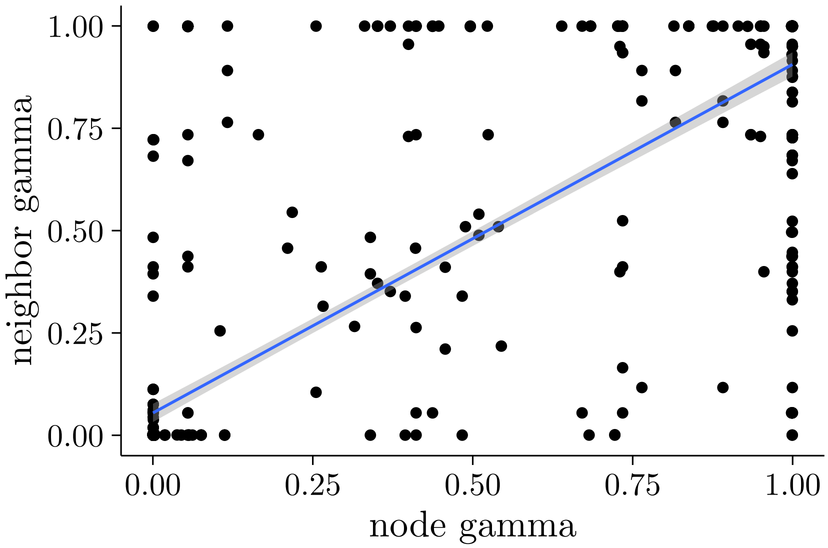

Figure S.3: γ values of a given node's neighbors, plotted against the node's own value of γ. The blue line indicates a linear regression.

	A	ambiguous	B
0	0	13	105
1	124	36	41

Table S.1: Contingency table of blockmodel partition (rows) against topic assignment (columns). $\chi^2 = 152$, $p = 1.3\text{e-}33$, $V = 0.69$.

blockmodel partition vs. discrete topic assignment. See table S.1.

There is a very strong correlation between the partitions and topic assignments. This can also be seen if we plot $\gamma_{a,j}$, the posterior distribution for topic A, against blockmodel partition assignment; see figure S.4.

However, it is difficult to map these topics to recognizable areas of toxicology research. Tables S.2 and S.3 give DOIs, titles, and γ values for the 10 articles mostly highly associated with topics A and B, respectively. Both tables contain papers on fundamental HTT research as well as applications of HTT to both human health and ecotoxicology. Similarly, table S.4 shows the terms most associated with the two topics. “Chemical,” “results,” “exposure,” and “data” appear in both lists, suggesting that the two topics substantially overlap. This lack of clarity and distinctness in the topics corresponds to the lack of stability observed above. It appears that the LDA model is unable to find substantive, consistent patterns in the distribution of terms.

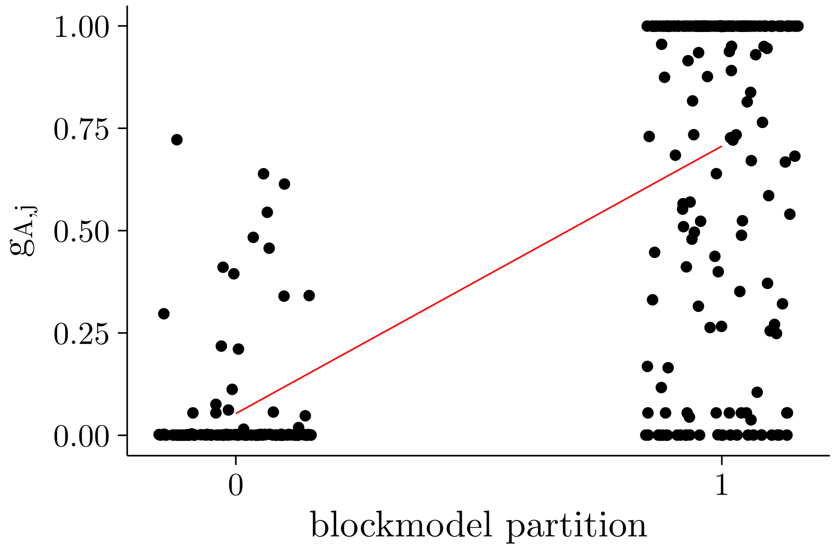

Figure S.4: γ value for topic A, plotted against blockmodel partition membership. The red line connects the mean values across the two partitions.

Conclusion

While this analysis finds very good concordance between the $k = 2$ topic model and the core nodes, in terms of both the citation network connecting these nodes by themselves and in terms of the blockmodel partition that they inherit from the larger citation network, text analysis of paper abstracts does *not* provide much insight into these topological features of this particular citation network.

Appendix: Reproducibility

System environment and package versions used to conduct this analysis:

```
## R version 3.3.1 (2016-06-21)
## Platform: x86_64-apple-darwin13.4.0 (64-bit)
## Running under: OS X 10.11.6 (El Capitan)
##
## locale:
## [1] en_US.UTF-8/en_US.UTF-8/en_US.UTF-8/C/en_US.UTF-8/en_US.UTF-8
##
## attached base packages:
## [1] parallel  stats     graphics  grDevices utils     datasets  methods
## [8] base
##
## other attached packages:
##  [1] cowplot_0.6.2     doParallel_1.0.10 iterators_1.0.8
```

```
##  [4] foreach_1.4.3     xtable_1.8-2      stringr_1.1.0
##  [7] reshape2_1.4.2    topicmodels_0.2-4 tidytext_0.1.2
## [10] knitr_1.14        igraph_1.0.1      httr_1.2.1
## [13] dplyr_0.5.0       purrr_0.2.2       readr_1.0.0
## [16] tidyr_0.6.0       tibble_1.2        ggplot2_2.1.0
## [19] tidyverse_1.0.0
##
## loaded via a namespace (and not attached):
##  [1] NLP_0.1-9         Rcpp_0.12.7       formatR_1.4
##  [4] plyr_1.8.4        tokenizers_0.1.4  tools_3.3.1
##  [7] digest_0.6.10     evaluate_0.9      gtable_0.2.0
## [10] nlme_3.1-128      lattice_0.20-34   filehash_2.3
## [13] Matrix_1.2-6      psych_1.6.9       DBI_0.5-1
## [16] hunspell_2.1      yaml_2.1.13       janeaustenr_0.1.4
## [19] stats4_3.3.1      grid_3.3.1        R6_2.2.0
## [22] foreign_0.8-67    rmarkdown_1.1     magrittr_1.5
## [25] codetools_0.2-14  modeltools_0.2-21 scales_0.4.0
## [28] SnowballC_0.5.1   htmltools_0.3.5   assertthat_0.1
## [31] mnormt_1.5-5      tikzDevice_0.10-1 colorspace_1.2-7
## [34] labeling_0.3      stringi_1.1.2     lazyeval_0.2.0
## [37] munsell_0.4.3     tm_0.6-2          slam_0.1-38
## [40] broom_0.4.1
```

References

1. Blei DM, Ng AY, Jordan MI. Latent Dirichlet allocation. The Journal of Machine Learning Research. 2003;3: 993–1022.

2. Mohr J, Bogdanov P. Introduction—Topic models: What they are and why they matter. Poetics. 2013;41: 545–69. doi:10.1016/j.poetic.2013.10.001

3. Hong L, Davidson B. Empirical study of topic modeling in twitter. Proceedings of the first workshop on social media analytics. ACM; 2010. pp. 80–88. doi:10.1145/1964858.1964870

4. Wallach H, Murray I, Salakhutdinov R, Mimno D. Evaluation methods for topic models. ICML '09 proceedings of the 26th annual international conference on machine learning. ACM; 2009. pp. 1105–12. doi:10.1145/1553374.1553515

5. Chang J, Boyd-Graber J, Wang C, Gerrish S, Blei DM. Reading tea leaves: How humans interpret topic models. Neural information processing systems 2009. 2009. Available: https://papers.nips.cc/paper/3700-reading-tea-leaves-how-humans-interpret-topic-models

6. Greene D, O'Callaghan D, Cunningham P. How many topics? Stability analysis for topic models. arXiv. 2014;1404.4606.

doi	title	gamma
10.1002/etc.2662	Development of an adverse outcome pathway for acetylcholinesterase inhibition leading to acute mortality	1.00
10.1093/toxsci/kfv196	Dose addition models based on biologically relevant reductions in fetal testosterone accurately predict postnatal reproductive tract alterations by a phthalate mixture in rats	1.00
10.1016/j.tox.2014.02.016	Environmentally relevant mixing ratios in cumulative assessments: A study of the kinetics of pyrethroids and their ester cleavage metabolites in blood and brain; and the effect of a pyrethroid mixture on the motor activity of rats	1.00
10.1371/journal.pone.0046579	Fishy Aroma of Social Status: Urinary Chemo-Signalling of Territoriality in Male Fathead Minnows (Pimephales promelas)	1.00
10.1016/j.aquatox.2015.12.024	Impaired anterior swim bladder inflation following exposure to the thyroid peroxidase inhibitor 2-mercaptobenzothiazole part I: Fathead minnow	1.00
10.1016/j.aquatox.2015.12.023	Impaired anterior swim bladder inflation following exposure to the thyroid peroxidase inhibitor 2-mercaptobenzothiazole part II: Zebrafish	1.00
10.1016/j.tiv.2013.02.012	Mechanism-based testing strategy using in vitro approaches for identification of thyroid hormone disrupting chemicals	1.00
10.1186/s13041-015-0099-9	Ontogeny of biochemical, morphological and functional parameters of synaptogenesis in primary cultures of rat hippocampal and cortical neurons	1.00
10.1021/es500016s	Perfluoroalkyl acid distribution in various plant compartments of edible crops grown in biosolids-amended soils	1.00
10.1021/acs.chemrestox.5b00024	Probe molecule (PrM) approach in adverse outcome pathway (AOP) based high-throughput screening (HTS): In vivo discovery for developing in vitro target methods	1.00

Table S.2: 10 articles most highly associated with topic A

doi	title	gamma
10.1007/s10669-014-9518-1	Data dialogues: critical connections for designing and implementing future nanomaterial research	1.00
10.1002/jctb.4695	Effect of membrane and process characteristics on cost and energy usage for separating alcohol-water mixtures using a hybrid vapor stripping-vapor permeation process	1.00
10.1021/ci300350r	Estimation of environment-related properties of chemicals for design of sustainable processes: Development of group-contribution+ (GC +) property models and uncertainty analysis	1.00
10.1021/ar5004219	Ferrates: Greener oxidants with multimodal action in water treatment technologies	1.00
10.1007/s11367-014-0726-1	Life cycle assessment for emerging materials: Case study of a garden bed constructed from lumber produced with three different copper treatments	1.00
10.1021/ar400309b	Microwave-assisted chemistry: Synthetic applications for rapid assembly of nanomaterials and organics	1.00
10.1039/c5cs00236b	Natural inorganic nanoparticles-formation, fate, and toxicity in the environment	1.00
10.1039/c3en00103b	Polymorph-dependent titanium dioxide nanoparticle dissolution in acidic and alkali digestions	1.00
10.1016/j.envres.2014.09.026	Serum concentrations of perfluorinated compounds (PFC) among selected populations of children and Adults in California	1.00
10.1186/1471-2148-14-7	Targeted approach to identify genetic loci associated with evolved dioxin tolerance in Atlantic Killifish (Fundulus heteroclitus)	1.00

Table S.3: 10 articles most highly associated with topic B

topic A	topic B
development	organic
model	society
human	nano
potential	risk
dose	surface
results	results
concentrations	tio
toxicity	data
study	water
based	study
effects	chemical
exposure	environmental
data	nanoparticles
chemical	exposure
chemicals	ag

Table S.4: Top 15 terms for topics A and B
